# Supplementary material for: Modular (de)construction of complex bacterial phenotypes by CRISPR/nCas9-assisted, multiplex cytidine base-editing
Source: Nat Commun. 2022 May 31;13:3026. doi: 10.1038/s41467-022-30780-z (PMC9156665; doi:10.1038/s41467-022-30780-z)
Supplement: Supplementary file 1 — Supplementary Information [file 41467_2022_30780_MOESM1_ESM.pdf]

Supplementary Information

Modular (de)construction of complex bacterial phenotypes by CRISPR/nCas9-asssisted, multiplex cytidine base-editing

Daniel C. Volke[a]†, Román A. Martino[b,c]†, Ekaterina Kozaeva[a], Andrea M. Smania[b,c] and Pablo I. Nikel[a]*

[a] The Novo Nordisk Foundation Center for Biosustainability, Technical University of Denmark, Kongens Lyngby, Denmark

[b] Departamento de Química Biológica Ranwel Caputto, Facultad de Ciencias Químicas, Universidad Nacional de Córdoba, Córdoba, Argentina

[c] Centro de Investigaciones en Química Biológica de Córdoba (CIQUIBIC), CONICET, Universidad Nacional de Córdoba, Córdoba, Argentina

† These authors contributed equally.

* Corresponding author e-mail: pabnik@biosustain.dtu.dk

Supplementary tables and figures

Supplementary Table 1. Bacterial strains and plasmids used in this study.

Strain	Relevant characteristics[a]	Reference or source
E. coli DH5α λ*pir*	Cloning host; F^{-} λ^{-} *endA1 glnX44*(AS) *thiE1 recA1 relA1 spoT1 gyrA96*(Nal^{R}) *rfbC1 deoR nupG* Φ80(*lacZ*ΔM15) Δ(*argF-lac*)*U169 hdR17*(r_K^{-} m_K^{+}), λ*pir* lysogen	Platt *et al.*[1]
P. aeruginosa PA14	Wild-type strain, clinical isolate from a human burn patient	Liberati *et al.*[2]
P. putida KT2440	Wild-type strain, derived from *P. putida* mt-2 strain[3] cured of the TOL plasmid pWW0	Bagdasarian *et al.*[4]
P. putida PCA	*P. putida* KT2440 engineered for PCA biosynthesis; *pcaG*^{Q81*}, *ppc*$^{Q74*\cdot W247*}$, *pykA*^{Q24*}, *pyk*$^{W52*\cdot Q59*}$, *aroF-I*^{D159N}, carrying plasmid pS2311·PCA; Km^{R}	This work
P. putida Δ*sthA*	*P. putida* KT2440 with clean *sthA* (*PP_2151*) deletion	Nikel *et al.*[5]
P. putida Δ*pntA*	*P. putida* KT2440 with clean *pntA* (*PP_0156*) deletion	Nikel *et al.*[5]
P. putida Δ*pntA* Δ*pntB*	*P. putida* KT2440 with clean *pntA* (*PP_0156*) and *pntB* (*PP_0155*) deletions	Nikel *et al.*[5]
P. putida TH	*P. putida* KT2440 with clean *sthA* (*PP_2151*), *pntA* (*PP_0156*) and *pntB* (*PP_0155*) deletions	Nikel *et al.*[5]
P. putida Δ*icd*	*P. putida* KT2440 with *icd* $^{Q78*\cdot Q139*}$	This work
P. putida LG	*P. putida* KT2440 with *icd*$^{Q78*\cdot Q139*}$, *ghrB*^{W124*} and *maeB*^{Q314*}	This work
P. putida Δ*icd* Δ*maeB* Δ*ghrB* Δ*pntA*	*P. putida* KT2440 with clean *pntA* (*PP_0156*) deletion and *icd*$^{Q78*\cdot Q139*}$, *ghrB*^{W124*} and *maeB*^{Q314*}	This work
P. putida Δ*icd* Δ*maeB* Δ*ghrB* Δ*pntA* Δ*pntB*	*P. putida* KT2440 with clean *pntA* (*PP_0156*) and *pntB* (*PP_0155*) deletions and *icd*$^{Q78*\cdot Q139*}$, *ghrB*^{W124*} and *maeB*^{Q314*}	This work
P. putida TH·UM	*P. putida* KT2440 Δ*sthA* *pntA*^{W238*} *pntB*^{Q117*} *pgi*-I$^{Q129*\cdot W229*}$ *pgi*-II^{Q129*}	This work
P. putida TH·UM·I	*P. putida* KT2440 Δ*sthA* *pntA*^{W238*} *pntB*^{Q117*} *pgi*-I$^{Q129*\cdot W229*}$ *pgi*-II^{Q129*} *icd*$^{Q78*\cdot Q139*}$	This work
P. putida TH·UM·LM	*P. putida* KT2440 Δ*sthA* *pntA*^{W238*} *pntB*^{Q117*} *pgi*-I$^{Q129*\cdot W229*}$ *pgi*-II^{Q129*} *ghrB*^{W124*} *maeB*^{Q314*}	This work
P. putida TH·UM·LM·I	NADHP-deficient strain for selection; *P. putida* KT2440 Δ*sthA* *pntA*^{W238*} *pntB*^{Q117*} *pgi*-I$^{Q129*\cdot W229*}$ *pgi*-II^{Q129*} *ghrB*^{W124*} *maeB*^{Q314*} *icd*^{Q78*}	This work

Plasmid	Relevant characteristics	Reference or source
pnCas9PA-BEC	Cytosine base-editing plasmid; *oriV*(pRO1600/ColE1), *sacB*; Gm^R	Chen *et al.*[6]
pSEVA434	Standard expression vector; *oriV*(pBBR1), *lacI*^{q}/P_{trc} cargo; Sm^R	Silva-Rocha *et al.*[7]
pS434·Cas6f	Derivative of vector pSEVA434 bearing the Cas6 endoribonuclease gene [*lacI*^{q}/P_{trc}→*cas6f*]; Sm^R	This work
pBEC2	Standard cytosine base-editing vector bearing the uracil glycosylase inhibitor (UGI) gene and the monomeric super-folder green fluorescent protein (msfGFP) gene; *oriV*(pRO1600/ColE1); Km^R	This work
pBEC6	Standard cytosine base-editing vector bearing the uracil glycosylase inhibitor (UGI) gene and the monomeric super-folder green fluorescent protein (msfGFP) gene; *oriV*(pRO1600/ColE1); Gm^R	This work
pMBEC2	Multiplex cytosine base-editing vector bearing the uracil glycosylase inhibitor (UGI) gene, the monomeric super-folder green fluorescent protein (msfGFP) gene, and the Cas6 endoribonuclease gene; *oriV*(pRO1600/ColE1); Km^R	This work
pMBEC4	Multiplex cytosine base-editing vector bearing the uracil glycosylase inhibitor (UGI) gene, the monomeric super-folder green fluorescent protein (msfGFP) gene, and the Cas6 endoribonuclease gene; *oriV*(pRO1600/ColE1); Sm^R	This work
pMBEC6	Multiplex cytosine base-editing vector bearing the uracil glycosylase inhibitor (UGI) gene, the monomeric super-folder green fluorescent protein (msfGFP) gene, and the Cas6 endoribonuclease gene; *oriV*(pRO1600/ColE1); Gm^R	This work
pMBEC8	Multiplex cytosine base-editing vector bearing the uracil glycosylase inhibitor (UGI) gene, the monomeric super-folder green fluorescent protein (msfGFP) gene, and the Cas6 endoribonuclease gene; *oriV*(pRO1600/ColE1); Ap^R	This work
pEX128·gRNA	Plasmid carrying template gRNA scaffold; Amp^R	This work
pSEVA621	Standard cloning vector; *oriV*(RK2); Gm^R	Silva-Rocha *et al.*[7]
pFDH	Derivative of vector pSEVA621 bearing an engineered formate dehydrogenase (FDH^e) gene from *Pseudomonas* sp. strain 101 under control of the P_{trc} promoter; Gm^R	This work
pSEVA2311	Standard expression vector; *oriV*(pBBR1), *chnrR*/P_{chnB} cargo; Km^R	Benedetti *et al.*[8]

pS2311·PCA	Derivative of vector pSEVA2311 bearing the *quiC*, *aroQ*, and *tktA* genes [*chnrR*/P_{chnB}→*quiC·aroQ·tktA*]; Km^R	This work
pMBEC6·*nicX*-Pos1	Derivative of vector pMBEC6 bearing a multiplex gRNA with *nicX-benA-gclR-glpR-nfxB* spacers; Gm^R	This work
pMBEC6·*nicX*-Pos2	Derivative of vector pMBEC6 bearing a multiplex gRNA with *benA-nicX-gclR-glpR-nfxB* spacers; Gm^R	This work
pMBEC6·*nicX*-Pos3	Derivative of vector pMBEC6 bearing a multiplex gRNA with *benA-gclR-nicX-glpR-nfxB* spacers; Gm^R	This work
pMBEC6·*nicX*-Pos4	Derivative of vector pMBEC6 bearing a multiplex gRNA with *benA-gclR-glpR-nicX-nfxB* spacers; Gm^R	This work
pMBEC6·*nicX*-Pos5	Derivative of vector pMBEC6 bearing a multiplex gRNA with *benA-gclR-glpR-nfxB-nicX* spacers; Gm^R	This work
pMBEC6·*nicX*-Pos5Cas6	Derivative of vector pMBEC6 bearing a multiplex gRNA with *benA-gclR-glpR-nfxB-nicX* spacers; Gm^R	This work
pMBEC6·PCA	Derivative of vector pMBEC6 bearing a multiplex gRNA with *pcaG*^{Q81*}, *pcaH*^{W16*}, *pps*$^{Q74*\cdot W247*}$, *pykA*^{Q24*}, *pyk*$^{W52*\cdot Q59*}$, and *aroF-I*^{D159N} spacers; Gm^R	This work
pMBEC6·Pgi	Derivative of vector pMBEC6 bearing a multiplex gRNA with *pgi*-I^{W229*}, and *pgi*-I-II^{Q129*} spacers; Gm^R	This work
pMBEC6·TCA	Derivative of vector pMBEC6 bearing a multiplex gRNA with *maeB*$^{Q152*\cdot Q314*}$, *ghrB*^{W124*}, and *ghrB2*^{W124*}, *icd*^{Q78*} and *icd*^{Q137*} spacers; Gm^R	This work
pMBEC6·*maeB-ghrB*	Derivative of vector pMBEC6 bearing a multiplex gRNA with *maeB*$^{Q152*\cdot Q314*}$, *ghrB*^{W124*}, and *ghrB2*^{W124*} spacers; Gm^R	This work
pMBEC6·*icd*	Derivative of vector pMBEC6 bearing a multiplex gRNA with *icd*^{Q78*} and *icd*^{Q137*} spacers; Gm^R	This work
pMBEC6·*Eco*	Derivative of vector pMBEC6 bearing a multiplex gRNA with *gshA*, *tnaA*, *speA*, *sdaA*, and *sdaB* spacers; Gm^R	This work
pMBEC6·PA14	Derivative of vector pMBEC6 bearing a multiplex gRNA with *PA14_02110*, *PA14_03790*, *PA14_04420*, *PA14_23130* and *PA14_53140* spacers; Gm^R	This work

[a] Antibiotic markers: Amp, ampicillin; Ap, apramycin; Gm, gentamicin; Km, kanamycin; Nal, nalidixic acid; and Sm, streptomycin.

Supplementary Table 2. Spacers used in this study.

Spacer name	Sequence (5'→3')	Target	Purpose
A1	ACA CAC ACC CAT GGG CTC CA	*PP_4206*	Characterization of the editing window
A2	CAC ACA CAC AAC TCC CCC TC	*PP_3676*	
C1	CCC CCC CCA CCT TTC ATC TG	*PP_3536*	
G1	GCG CGC GCG CCT GCT GGC CC	*PP_4238*	
G2	TGC GCG CGC GCC TGC TGG CC	*PP_4238*	
T1	TCT CTC TCG TGT ATT GTC GG	Intergenic	
T2	ATC TCT CTC TGC CTC TTC TT	*PP_1539*	
benA	CGA TCA GTC GCC TGA AGG CC	*PP_3161*	Determination of editing efficiency through phenotypic analysis of *P. putida* *nicX*^{W187*} edit
gclR	CCT GCA CGA GTC GGC AAG GC	*PP_4283*	
glpR	CGC CGA GAT GGT GGT GTC GT	*PP_1074*	
nfxB	CCA CCC CCG AGC CAC ATT GA	*PP_2820*	
nicX	GTC CCA GCG CCC CGG TTC GT	*PP_3945*	
pcaG	GTA CCA GGA TGC CTA CAA CC	*PP_4655*	Engineering of a protocatechuic acid production strain
pcaH	TAG GGT GCC AGT TGC GAT CA	*PP_4656*	
ppc1	CAC CCA TCC ACG AGG CAA AG	*PP_1505*	
ppc2	CTT CAA CCA GTT CCT CAA CC	*PP_1505*	
pykA1	ATC GAA CAA CTG ATC CTC GC	*PP_1362*	
pykA2	TAC CAG CCA GGC GCT GCC GC	*PP_1362*	
pyk1	CAA CAG CTG AAC TAC CCG CT	*PP_4301*	
pyk2	GCG GAT CCA CTG GTA GCG CA	*PP_4301*	
aroF-I^{D159N}	CGG GTC GAG CGC TTC GGT GG	*PP_2324*	
icd1	AGC CAC CCA GGT GTA TGA CC	*PP_4011*	Construction of a NADPH-depleted *P. putida* strain
icd2	GTT CCA GGG TGT GCC AAG CC	*PP_4011*	
maeB1	GGA TCG AAC AGT GCG ACA TC	*PP_5085*	
maeB2	AGT ACG TTG TTG ACC TGG TT	*PP_5085*	
ghrB	CTT GGT CCA GGC ATC CAG CT	*PP_1261*	
pgi-I·II	AAC CAG ATT ACC GAA CTG GT	*PP_1808*<br>*PP_4701*	
pgi-I	TGG CCG CGC GGA CCT GGT AC	*PP_1808*	
gshA	AAT CCA TTT GTG CGT CAG TG	*ECD_02543*	Multiplex editing in *E. coli* BL21
tnaA	AGG TGG TCA GCC GGT TTC AC	*ECD_03592*	
speA	AAT ATT CAG TGC TTC GAC GT	*ECD_02768*	
sdaA	GGC ACA GGG ACG GCA TGA AG	*ECD_01784*	
sdaB	ATT CAG GAT GTG AAT ACT CA	*ECD_02642*	

PA14_02110	CCA CGG CCA GAG CTA CGT GC	*PA14_02110*	Multiplex editing in *P. aeruginosa PA14*
PA14_03790	CGG CCA CCA GGT CGG CGA CC	*PA14_03790*	
PA14_04420	GGC CAG TGG CCT CTA CCG AA	*PA14_04420*	
PA14_23130	AGT CCG CCA ACT GAT CCC GC	*PA14_23130*	
PA14_53140	CGT CCA GTT GGT CAT CTG CT	*PA14_53140*	

Supplementary Table 3. Oligonucleotides used in this study.[a]

Oligonucleotide		**Sequence** (5'→3')	**Purpose**
**Number**	**Name**		
1	pR1	TTA GAC TCT CGT TTG GAT TGC	Sequencing multiplex-gRNA assembly in pMBEC vectors
2	pR2	AAC GAA ATG ACA GAG CGT GC	
3	pR3	ACT GCC GGT TCT CCG AAT TGC AG	
4	UGI-BsaI-nCas9-U-F†	AGG TGA CUC CGG CGG CTC CAC CAA CCT GTC CGA CAT CAT CGA GAA GGA AAC C	Fusion of UGI and nCas9 genes in vector pBEC6
5	UGI-BsaI-nCas9-U-R†	ACA GCA UCT TGA TCT TGT TCT CGC CGT TGG A	
6	PABEC-UGI-U-F†	ATG CTG UGA ACT TCG GAA TAG GAA CTT CA	
7	PABEC-UGI-U-R†	AGT CAC CUC CTA GCT GAC TCA AAT CA	
8	*msfGFP*-BsaI-F	ATG TGT GGC GAG ACC AGG AGG AAA AAC ATA TGC GTA AAG GTG	Introducing *msfGFP* between *Bsa*I sites for gRNA cloning
9	*msfGFP*-BsaI-R	TCT AAA ACT GAG ACC TTA TTT GTA GAG TTC ATC CAT GCC GTG	
10	Cas6-RBS-EcoRI-F†	GCG CGA ATT CAA GCC GAA GAG AAG TAA GCC ATG G	Cloning *cas6f* (*PA14_33300*)
11	Cas6-BamHI-R	TCC TGG ATC CTC AGA ACC AGG GAA CGA AAC CTC C	
12	LacO-Cas6-XbaI-F	ATG GTC TAG ATT GAC AAT TAA TCA TCC GGC TCG TAT AAT G	Cloning P_{trc}→*cas6f* into pBEC vectors
13	Cas6-XhoI-R	ATG CCT CGA GTC AGA ACC AGG GAA CGA AAC CTC	
14	*quiC*-RBS-BsaI-F	ATC GAG GTC TCC AGG AGG AAA AAC ATA TGC AGC GTT CGA TCG CT AC	Amplification of *quiC* (*PP_2554*)
15	*quiC*-BsaI-R	ATC GAG GTC TCC CTC CTC TAC AGC ACC GGC TTG CGC G	
16	*aroQ*-RBS-BsaI-F	ATC GAG GTC TCC GGA GGA AAA ACA TAT GGC AAC GCT ACT GGT GCT	Amplification of *aroQ*-I (*PP_0560*)
17	*aroQ*-BsaI-R	ATC GAG GTC TCC CCT CCT TCA TTT GGG CTG TGC GTT GGC	
18	*tktA*-RBS-BsaI-F	ATC GAG GTC TCC GAG GAA AAA CAT ATG TCC TCA CGT AAA GAG CTT GCC	Amplification of *tktA* (*PP_4965*)
19	*tktA*-BsaI-R	ATC GAG GTC TCC CCT CCT TCA TTT GGG CTG TGC GTT GGC	
20	pSEVA2311-BsaI-F	ATC GAG GTC TCC GGA TCC TCT AGA GTC GAC CTG C	Amplification of vector pS2311 for inserting *quiC*, *aroQ* and *tktA*
21	pSEVA2311-BsaI-R	ATC GAG GTC TCC TCC TCC GGG TAC CGA GCT CGA AT	

22	*Ptrc-FDH*-U-F†	ATC CTT GAC AAU TAA TCA TCC GGC TCG TAT	Construction of plasmids for expression of $P_{trc\rightarrow}$FDH[e]
23	*Ptrc-FDH*-U-R†	ACT CTA GAU TAA ACA GCT TTT TTG AAT TTA GC	
24	pSEVA621-U-F†	ATC TAG AGU CGA CCT GCA GGC ATG CAA	
25	pSEVA621-U-R†	ATT GTC AAG GAU CCC CGG GTA CCG AGC TCG AA	
26	*nicX*-Seq-F	CCC AGC GTT TGA AGG CCA AGG T	Sequencing of *nicX*
27	*nicX*-Seq-R	ATT TCC AGG GTG ATG CGC TCG C	
28	*benA*-F	atc gAG GTC TCC CGA TCA GTC GCC TGA AGG CCg ttt tag agc tag aaa tag c	Constructing pMBEC derivatives to target *nicX* at different gRNA positions
29	*benA*-R	atc gAG GTC TCC GGA Ctt tct tag ctg cct ata cgg	
30	*benA*-pSEVA-F	atc gAG GTC TCC GTG GCG ATC AGT CGC CTG AAG GCC gtt tta gag cta gaa ata gc	
31	*benA*2-R	atc gAG GTC TCC CAG Gtt tct tag ctg cct ata cgg	
32	*gclR*-F	atc gAG GTC TCC CCT GCA CGA GTC GGC AAG GCg ttt tag agc tag aaa tag c	
33	*gclR*-R	atc gAG GTC TCC GGC Gtt tct tag ctg cct ata cgg	
34	*glpR*-F	atc gAG GTC TCC CGC CGA GAT GGT GGT GTC GTg ttt tag agc tag aaa tag c	
35	*glpR*-R	atc gAG GTC TCC GTG Gtt tct tag ctg cct ata cgg	
36	*nfxB*-F	atc gAG GTC TCC CCA CCC CCG AGC CAC ATT GAg ttt tag agc tag aaa tag c	
37	*glpR_nfxB*-R	atc gAG GTC TCC AAA CTC AAT GTG GCT CGG GGG TGG ttt ctt agc tgc cta tac gg	Constructing pMBEC derivatives to target *nicX* at different gRNA positions
38	*nicX2*-F	atc gAG GTC TCC GTC CCA GCG CCC CGG TTC GTg ttt tag agc tag aaa tag c	
39	*nfxB_nicX*-R	atc gAG GTC TCC AAA CAC GAA CCG GGG CGC TGG GAC ttt ctt agc tgc cta tac gg	
40	*nicX*-pSEVA-F	atc gAG GTC TCC GTG GGT CCC AGC GCC CCG GTT CGT gtt tta gag cta gaa ata gc	
41	*nicX*-R	atc gAG GTC TCC ATC Gtt tct tag ctg cct ata cgg	
42	*PP_benA*-F	atc gAG GTC TCC GTG GCG ATC AGT CGC CTG AAG GCC	
43	*PP_nicXB*-F	atc gAG GTC TCC TCG Tgt ttt aga gct aga aat agc	
44	*PP_nicX1*-R	atc gAG GTC TCC ACG AAC CGG GGC GCT GGG AC	
45	*PP_nicX2*-R	atc gAG GTC TCC AAA Ctt tct tag ctg cct ata cgg	
46	gRNAPosition1-GG-R	atc gAG GTC TCC acc tTT AGC TGC CTA TAC GGC AGT	Standard Golden Gate oligonucleotides for assembly of multiple
47	gRNAPosition2-GG-R	atc gAG GTC TCC ccg cTT AGC TGC CTA TAC GGC AGT	

48	gRNAPosition3-GG-R	atc gAG GTC TCC aca aTT AGC TGC CTA TAC GGC AGT	gRNAs in pMBEC vectors
49	gRNAPosition4-GG-R	atc gAG GTC TCC aac aTT AGC TGC CTA TAC GGC AGT	
50	gRNAPosition5-GG-R	atc gAG GTC TCC gaa aTT AGC TGC CTA TAC GGC AGT	
51	gRNAPosition6-GG-R	atc gAG GTC TCC caa gTT AGC TGC CTA TAC GGC AGT	
52	gRNAPosition7-GG-R	atc gAG GTC TCC gca cTT AGC TGC CTA TAC GGC AGT	
53	gRNAPosition8-GG-R	atc gAG GTC TCC tag aTT AGC TGC CTA TAC GGC AGT	
54	gRNAPosition9-GG-R	atc gAG GTC TCC aaa tTT AGC TGC CTA TAC GGC AGT	
55	gRNAPosition10-GG-R	atc gAG GTC TCC gga gTT AGC TGC CTA TAC GGC AGT	
56	gRNAPosition11-GG-R	atc gAG GTC TCC tga cTT AGC TGC CTA TAC GGC AGT	
57	gRNAPosition12-GG-R	atc gAG GTC TCC tcc cTT AGC TGC CTA TAC GGC AGT	
58	*pcaG*1-F	ATC GAG GTC TCC GTG Ggt acc agg atg cct aca acc GTT TTA GAG CTA GAA ATA GC	Spacer for *pcaG* (*PP_4655*)
59	*pcaH*2-F	ATC GAG GTC TCC AGG Tta ggg tgc cag ttg cga tca GTT TTA GAG CTA GAA ATA GC	Spacer for *pcaH* (*PP_4656*)
60	*ppc-I*3-F	ATC GAG GTC TCC GCG Gca ccc atc cac gag gca aag GTT TTA GAG CTA GAA ATA GC	Spacer 1 for *ppc* (*PP_1505*)
61	*ppc-II*-F	ATC GAG GTC TCC TTG Tct tca acc agt tcc tca acc GTT TTA GAG CTA GAA ATA GC	Spacer 2 for *ppc* (*PP_1505*)
62	*pykA-I*5-F	ATC GAG GTC TCC TGT Tat cga aca act gat cct cgc GTT TTA GAG CTA GAA ATA GC	Spacer 1 for *pykA* (*PP_1362*)
63	*pykA-II*6-F	ATC GAG GTC TCC TTT Cta cca gcc agg cgc tgc cgc GTT TTA GAG CTA GAA ATA GC	Spacer for *pykA* (*PP_1362*)
64	*pyk-I*7-F	ATC GAG GTC TCC CTT Gca aca gct gaa cta ccc gct GTT TTA GAG CTA GAA ATA GC	Spacer 1 for *pyk* (*PP_4301*)
65	*pyk-II*8-F	ATC GAG GTC TCC GTG Cgc gga tcc act ggt agc gca GTT TTA GAG CTA GAA ATA GC	Spacer 2 for *pyk* (*PP_4301*)
66	*aroF-I*^{D159N}9-F	ATC GAG GTC TCC TCT Acg ggt cga gcg ctt cgg tgg GTT TTA GAG CTA GAA ATA GC	Spacer for *aroF-I* (*PP_2324*)
67	*pcaG*-Seq-F	TGA TGT GCG GCG CCA TCG GC	Sequencing *pcaG* and *pcaH*
68	*pcaH*-Seq-R	GCC ACG CCC AGG CGT TCT CAC	

69	*ppc*-Seq-F	TCC GCC ACA GTG CCA AGG CC	Sequencing *ppc*
70	*ppc*-Seq-R	TGC GCA GGG TGT CGT TGG CC	
71	*pykA*-Seq-F	GCG GCG GGC GTG CTG GAT GA	Sequencing *pykA*
72	*pykA*-Seq-R	GCA TGA GCA TCC GCC GCA CC	
73	*pyk*-Seq-F	CTT GCC ACC CTT GGC CCT GC	Sequencing *pyk*
74	*pyk*-Seq-R	CCA GCT CCA GGC CAA AGG CC	
75	*aroF*-Seq-F	GAT GCC CAG GAA GCG GTG CGG	Sequencing *aroF-I*
76	*aroF*-Seq-R	CGC CAA GGC CCT GCA GAC CG	
77	*pgi-I*-Seq-F	AGA CGG TTC CAC CCA AGG CC	Sequencing *pgi-I*
78	*pgi-I*-Seq-R	CGG GCC AGT ATC AAG CAG GG	
79	*pgi-II*-Seq-F	GAT CAA CTA CTT CCG CGG CC	Sequencing *pgi-II*
80	*pgi-II*-Seq-R	GCG CAA TTC GGC TTC GGC TT	
81	*icd*-Seq-F	GCC AAA GCC TGA GCT GCT TAC C	Sequencing *icd*
82	*icd*-Seq-R	CGG CGT CGA TGT TTC GCC GG	
83	*maeB*-Seq-F	TCA TCC ACG CTG GTC AGC GG	Sequencing *maeB*
84	*maeB*-Seq-R	CGC CGC TCT CGA ATA TCA CGC	
85	*ghrB*-Seq-F	CAG CCC TTC CAC ACC TGC G	Sequencing *ghrB*
86	*ghrB*-Seq-R	GAC CGT CCT GGC CTT CAG CC	
87	*maeB1*Pos1-GG-F	ATC GAG GTC TCC GTG GGA TCG AAC AGT GCG ACA TCC GTT TTA GAG CTA GAA ATA GC	Spacer for *maeB* (*PP_5085*)
88	*maeB2*Pos2-GG-F	ATC GAG GTC TCC AGG TAA CCA GGT CAA CAA CGT ACT GTT TTA GAG CTA GAA ATA GC	
89	*ghrB1*Pos3-GG-F	ATC GAG GTC TCC GCG GCT TGG TCC AGG CAT CCA GCT GTT TTA GAG CTA GAA ATA GC	Spacer for *ghrB* (*PP_1261*)
90	*ghrB2*Pos4-GG-F	ATC GAG GTC TCC TTG TGG TCC AGG CAT CCA GCT CGG GTT TTA GAG CTA GAA ATA GC	
91	*pgi-I*·IIPos5-GG-F	ATC GAG GTC TCC TGT TAA CCA GAT TAC CGA ACT GGT GTT TTA GAG CTA GAA ATA GC	Spacers for *pgi-I* (*PP_1808*) and *pgi-II* (*PP_4701*)
92	*pgi-I*·IIPos6-GG-F	ATC GAG GTC TCC TTT CGT ACC AGG TCC GCG CGG CCA GTT TTA GAG CTA GAA ATA GC	
93	*pgi-II*2-R	ATC GAG GTC TCC AAA CTG GCC GCG CGT ACC TGG TAC TTT CTT AGC TGC CTA TAC GG	
94	*icd1*Pos1-GG-F	ATC GAG GTC TCC GTG GAG CCA CCC AGG TGT ATG ACC GTT TTA GAG CTA GAA ATA GC	Spacer for *icd* (*PP_4011*)
95	*icd2*Pos2-GG-F	ATC GAG GTC TCC GTT CCA GGG TGT GCC AAG CCG TTT TAG AGC TAG AAA TAG C	
96	*gshA*Pos1-GG-F	ATC GAG GTC TCC GTG GAA TCC ATT TGT GCG TCA GTG GTT TTA GAG CTA GAA ATA GC	Spacer for *gshA* (*ECD_02543*)
97	*tnaA*Pos2-GG-F	ATC GAG GTC TCC AGG TAG GTG GTC AGC CGG TTT CAC GTT TTA GAG CTA GAA ATA GC	Spacer for *tnaA* (*ECD_03592*)
98	*speA1*Pos3-GG-F	ATC GAG GTC TCC GCG GAA TAT TCA GTG CTT CGA CGT GTT TTA GAG CTA GAA ATA GC	Spacers for *speA* (*ECD_02768*)
99	*speA2*Pos4-GG-F	ATC GAG GTC TCC TTG TGG CAC AGG GAC GGC ATG AAG GTT TTA GAG CTA GAA ATA GC	

100	*sdaB*Pos5-GG-F	ATC GAG GTC TCC TGT TAT TCA GGA TGT GAA TAC TCA GTT TTA GAG CTA GAA ATA GC	Spacer for *sdaB* (*ECD_02642*)
101	*gshA*-Seq-F	TTT GCA TCA GCG CGC CGT AG	Sequencing *gshA*
102	*gshA*-Seq-R	TAC AGC GTG GGC TGG AGC GC	
103	*tnaA*-Seq-F	GCC TTC GAT ACG GGC GTG CG	Sequencing *tnaA*
104	*tnaA*-Seq-R	TGG CGG ACA TCG CCA GCA TA	
105	*speA*-Seq-F	ACC GTC GGA TGC GGC AGA CC	Sequencing *speA*
106	*speA*-Seq-R	GCA CGT CTG GCT TCG CAG GG	
107	*sdaA*-Seq-F	GGG ATT GGT CCC TCA TCT TCC C	Sequencing *sdaA*
108	*sdaA*-Seq-R	TTT GAA CGG ATA CGG CAC GC	
109	*sdaB*-Seq-F	GGC CAC CAC ACT GAT ATC GCC	Sequencing *sdaB*
110	*sdaB*-Seq-R	GGC CAG AGA GTG ACA GCC CG	
111	*nfx*-Seq-F	GGG ATC GAT GCC CTG GGG	Sequencing *nfxB*
112	*nfx*-Seq-R	CCG CAC GAT ATG CAG TGT GG	
113	*benA*-Seq-F	CCA GCC GTC TCC CTA ACC TG	Sequencing *benA*
114	*benA*-Seq-R	TGC TGC AGC ACG AAC AGG CC	
115	*gclR*-Seq-F	GGA AGG GCT GCC TTG CAG C	Sequencing *gclR*
116	*gclR*-Seq-R	TCT TCG TGC CTC GAG AGC CC	
117	*glpR*-Seq-F	GTC TGG CGG CAT GCA CCC AC	Sequencing *glpR*
118	*glpR*-Seq-R	TTG TCC GGC AGC CCC AAA GG	

[a] Oligonucleotides designed for *USER* cloning are indicated with a † symbol. Synthetic ribosome binding sites (*RBS*) are underlined (__), restriction enzyme recognition sites are indicated by a broken underscore line (.....), spacer sequences are displayed in lower case.

Supplementary Table 4. Polymorphisms detected in engineered strains.

Strain	Mutations compared to reference genome	Mutations present in negative control (pSEVA631)	Unique mutations	Intended mutations	Off-target mutation/random mutations
P. putida pSEVA631	72	64	8	0	8
P. putida pnCas9-6	81	67	14	0	14
P. putida pnCas9-BEC6-C	86	67	19	1	18
P. putida pBEC6-C	98	63	35	2	33
P. putida PCA	94	61	33	10	23
P. putida TH·UM·LM·I	199	65	134	40	94

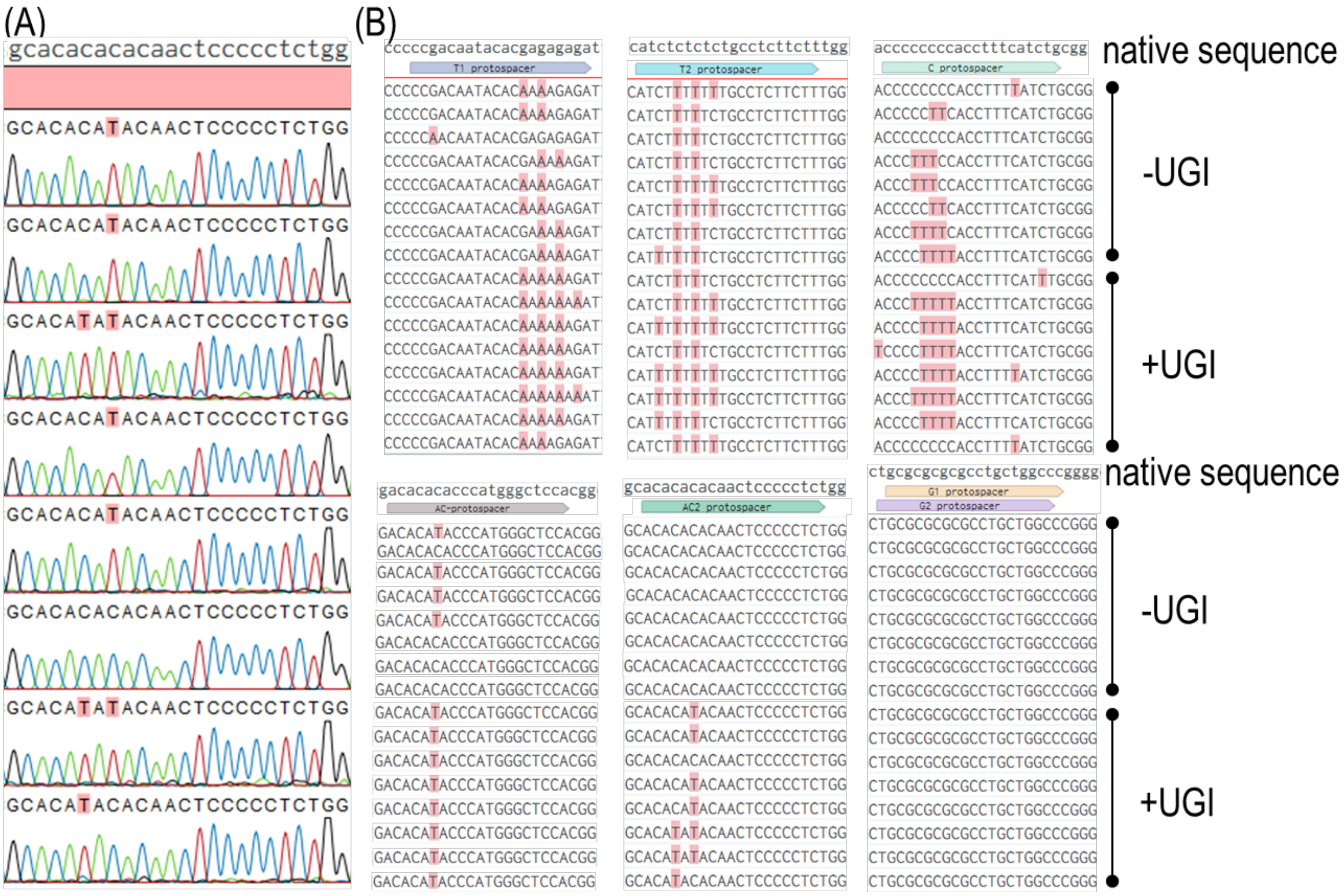

Supplementary Figure 1 | Screening of the editing window in base-editing experiments. **(A)** Chromatograms of adenosine-rich protospacer region in edited clones. The non-edited protospacer sequence is shown in the top line. **(B)** Experimental dataset from determining the base-editing window of the CBE constructed in this work. The upper eight sequences correspond to the editor without UGI, while the lower eight to the base editor added with UGI. The exact position of the protospacer is indicated in each case.

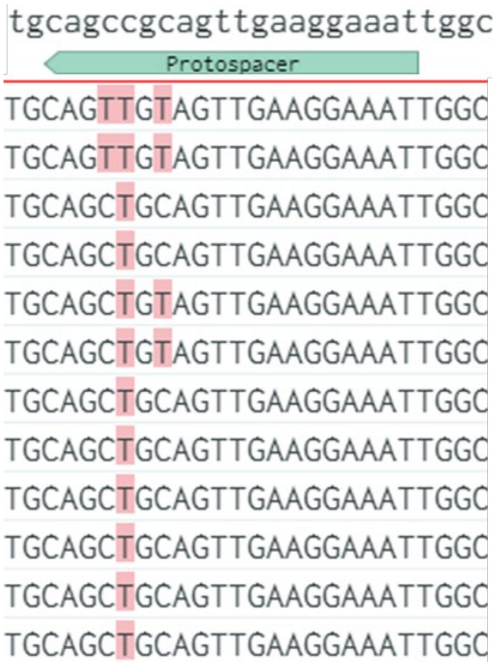

Supplementary Figure 2 | Base-editing in the presence of a proceeding guanidine. Editing was carried out in the *mutS* gene of *P*. *putida* KT2440. The upper sequence represents the non-edited protospacer sequence, and the protospacer sequence is highlighted. Editing experiments were carried out for 48 h.

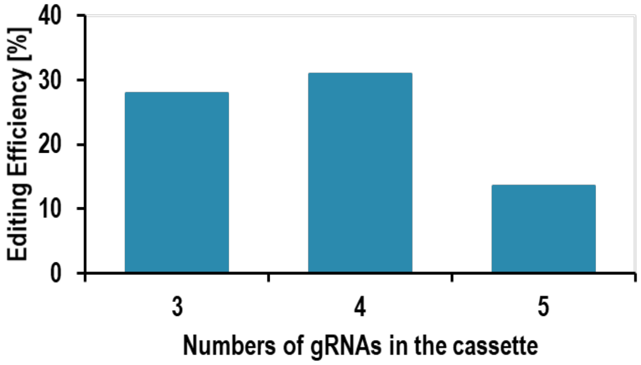

Supplementary Figure 3 | Editing efficiency of the last gRNA in a multiplex cluster. Clusters with three, four and five gRNAs were constructed, and the editing efficacy of the last gRNA was determined through the NicX-dependent scoring assay. Mean values from three independent experiments are presented.

Supplementary Methods

Detailed protocol for cloning multiple gRNAs in pMBEC plasmids

Spacers design

Design spacers for the genes of interest employing the CRISPY-web service[9]. Choose either spacers for multiple genes or multiple spacers for one gen. Select the protospacer sequences for C to T editing according to the following criteria:

- The "C" site to be edited should be in positions 3 to 9 in the suggested spacer sequence.
- For maximum efficiency, there should not be a "G" in front of the "C" to be edited.
- Preferably, the introduced *STOP* codon should be introduced towards the 5' end of the CDS, checking that there are no in-phase *START* codons downstream that could allow the translation of a truncated but potentially functional protein.

Oligo designer to introduce the spacers in pMBEC plasmids

Download the GoldenGate_PrimerDesigner sheet from http://www.sem-cfb.com/bioinfo.html. Introduce the number of spacers to clone into the pMBEC plasmid, followed by indicating the name of the genes and the sequences for the spacers in the corresponding field boxes. The sheet will generate forward primers with the specific standardized overhangs for each position as is shown in **Supplementary Methods Table 1**. The use of standard overhangs increases the efficiency of Golden Gate assembly and makes it independent of the number of spacers to be cloned[10]. Furthermore, the sheet file will generate a reverse primer for the last spacer. The first forward oligonucleotide will always have a fixed *GTGG* overhang preceding the first spacer and the last generated reverse oligo will include the reverse complementary sequence for the final spacer and a fixed *CAAA* overhang for cloning into the pMBEC backbone.

Supplementary Methods Table 1| Fragment overhangs created in GG cloning.

Position	Overhang sequence
1	GTGG
2	AGGT
3	GCGG
4	TTGT
5	TGTT
6	TTTC
7	CTTG
8	GTGC
9	TCTA
10	ATTT
11	CTCC
12	GTCA
13	GGGA

gRNA amplification by PCR from plasmid pEX128-gRNA

For each pair of oligonucleotides (i.e., forward and standard-reverse), perform a PCR amplification with Phusion High-Fidelity DNA polymerase (Thermo Fisher Scientific, USA) using the pEX128·gRNA plasmid **(Supplementary Table S1**) as template as indicated in **Supplementary Methods Table 2** and **3**.

Supplementary Methods Table 2| Reaction mixture for amplification of gRNAs.

Component	Volume
Phusion HS (TFS)	0.1 μL
DMSO	0.3 μL
dNTPs [10 mM]	0.2 μL
5× Buffer HS	2 μL
pEX128·gRNA plasmid [10 ng/μL]	1 μL
Oligo forward [10 nM]	0.5 μL
Oligo reverse [10 nM]	0.5 μL
H_2O	5.4 μL
Total volume	10 μL

Supplementary Methods Table 3| Thermocycler program for amplification of gRNAs.

Step	Temperature	Time	Cycle
1	94°C	5 min	1
2	94°C	20 s	30
	60°C	20 s	
	72°C	15 s	
3	72°C	2 min	1

Golden Gate assembly

After diluting the PCR products obtained in the previous step 1:50 with water (**Supplementary Methods Table 4**), prepare the Golden Gate reaction (**Supplementary Methods Table 5**).

Supplementary Methods Table 4| Dilution of PCR products.

PCR product	Volume
Fragment 1	1 μL
Fragment 2	1 μL

Fragment N_n	1 μL
H_2O	To complete 50 μL

Supplementary Methods Table 5|Golden Gate reaction mix.

Component	Volume
Diluted PCR mix	2 μL
pMBEC [100 ng/μL]	1 μL
10× T4 ligase buffer	1 μL
T4 ligase buffer (TFS)	0.5 μL
FastDigest EcoR1I (TFS)	0.5 μL
H_2O	6 μL
Total volume	10 μL

Set the thermocycler with the program (**Supplementary Methods Table 6**).

Supplementary Methods Table 6|Thermocycler program for Golden Gate reaction.

Step	Temperature	Time	Cycle
1	37°C	5 min	20-40
	16°C	5 min	
2	37°C	10 min	1
3	80°C	10 min	1

The number of cycles depends on the number of fragments to be assembled. In general, the efficiency of the reaction increases with the number of cycles, but for single-spacer insertion, a low number of cycles is sufficient.

Transformation of E. coli competent cells and selection of positive colonies

Transform *E. coli* chemical competent cells with the reaction mixture, plate the mixture onto LB medium plates supplemented with appropriate antibiotics and incubate for 24 h at 37°C. On the next day, check colonies for msfGFP signal (i.e., correct plasmid constructs should not display fluorescence). Select these non-fluorescent colonies and confirm genotypes by colony PCR with *OneTaq* Quick-Load 2× Master Mix (New England Biolabs, USA) with the primer pair pR1 (5'-TTA GAC TCT CGT TTG GAT TGC-3') and pR3 (5'-ACT GCC GGT TCT CCG AAT TGC AG-3'). Next, send the PCR products to be sequenced with primer pR3 to further corroborate clone identity and correctness by Sanger sequencing.

References

1. Platt, R., Drescher, C., Park, S.K. & Phillips, G.J. Genetic system for reversible integration of DNA constructs and *lacZ* gene fusions into the *Escherichia coli* chromosome. *Plasmid* **43**, 12-23 (2000).
2. Liberati, N.T. et al. An ordered, nonredundant library of *Pseudomonas aeruginosa* strain PA14 transposon insertion mutants. *Proc. Natl. Acad. Sci. USA* **103**, 2833-2838 (2006).
3. Worsey, M.J. & Williams, P.A. Metabolism of toluene and xylenes by *Pseudomonas putida* (*arvilla*) mt-2: evidence for a new function of the TOL plasmid. *J. Bacteriol.* **124**, 7-13 (1975).
4. Bagdasarian, M. et al. Specific purpose plasmid cloning vectors. II. Broad host range, high copy number, RSF1010-derived vectors, and a host-vector system for gene cloning in *Pseudomonas*. *Gene* **16**, 237-247 (1981).
5. Nikel, P.I., Pérez-Pantoja, D. & de Lorenzo, V. Pyridine nucleotide transhydrogenases enable redox balance of *Pseudomonas putida* during biodegradation of aromatic compounds. *Environ. Microbiol.* **18**, 3565-3582 (2016).
6. Chen, W. et al. CRISPR/Cas9-based genome editing in *Pseudomonas aeruginosa* and cytidine deaminase-mediated base editing in *Pseudomonas* species. *iScience* **6**, 222-231 (2018).
7. Silva-Rocha, R. et al. The Standard European Vector Architecture (SEVA): a coherent platform for the analysis and deployment of complex prokaryotic phenotypes. *Nucleic Acids Res.* **41**, D666-D675 (2013).
8. Benedetti, I., de Lorenzo, V. & Nikel, P.I. Genetic programming of catalytic *Pseudomonas putida* biofilms for boosting biodegradation of haloalkanes. *Metab. Eng.* **33**, 109-118 (2016).
9. Blin, K., Pedersen, L.E., Weber, T. & Lee, S.Y. CRISPy-web: An online resource to design sgRNAs for CRISPR applications. *Synth Syst Biotechnol* **1**, 118-121 (2016).
10. Pryor, J.M. et al. Enabling one-pot Golden Gate assemblies of unprecedented complexity using data-optimized assembly design. *PLoS One* **15**, e0238592 (2020).
