# Supplementary material for: Modular (de)construction of complex bacterial phenotypes by CRISPR/nCas9-assisted, multiplex cytidine base-editing
Source: Nat Commun. 2022 May 31;13:3026. doi: 10.1038/s41467-022-30780-z (PMC9156665; doi:10.1038/s41467-022-30780-z)
Supplement: Supplementary file 2 — Reporting Summary [file 41467_2022_30780_MOESM2_ESM.pdf]

nature portfolio

Corresponding author(s): Pablo Ivan Nikel
Last updated by author(s): May 15, 2022

Reporting Summary

Nature Portfolio wishes to improve the reproducibility of the work that we publish. This form provides structure for consistency and transparency in reporting. For further information on Nature Portfolio policies, see our Editorial Policies and the Editorial Policy Checklist.

Statistics

For all statistical analyses, confirm that the following items are present in the figure legend, table legend, main text, or Methods section.

n/a	Confirmed	
☐	☒	The exact sample size (*n*) for each experimental group/condition, given as a discrete number and unit of measurement
☐	☒	A statement on whether measurements were taken from distinct samples or whether the same sample was measured repeatedly
☐	☒	The statistical test(s) used AND whether they are one- or two-sided<br>*Only common tests should be described solely by name; describe more complex techniques in the Methods section.*
☒	☐	A description of all covariates tested
☒	☐	A description of any assumptions or corrections, such as tests of normality and adjustment for multiple comparisons
☐	☒	A full description of the statistical parameters including central tendency (e.g. means) or other basic estimates (e.g. regression coefficient) AND variation (e.g. standard deviation) or associated estimates of uncertainty (e.g. confidence intervals)
☐	☒	For null hypothesis testing, the test statistic (e.g. *F*, *t*, *r*) with confidence intervals, effect sizes, degrees of freedom and *P* value noted<br>*Give P values as exact values whenever suitable.*
☒	☐	For Bayesian analysis, information on the choice of priors and Markov chain Monte Carlo settings
☒	☐	For hierarchical and complex designs, identification of the appropriate level for tests and full reporting of outcomes
☒	☐	Estimates of effect sizes (e.g. Cohen's *d*, Pearson's *r*), indicating how they were calculated

Our web collection on statistics for biologists contains articles on many of the points above.

Software and code

Policy information about availability of computer code

Data collection	A custom-made code (Supplemental Data 1) written in Python (3.7.4) with Biopython (1.76) and available together with this submission was used in Spyder 5.1.5 to find all putative base editing sites in genomes (Figure 1); PatScanUI (https://patscan.secondarymetabolites.org/) was used to find protospacers with specific patterns; growth data was collected in a SynergyMX microtiter plate reader or ELx808 microtiter plate reader; DNA concentration was determined in a NanoDrop ND-1000 UV-Vis Spectrophotometer,
Data analysis	Chromeleon 7 (7.2.9) was used to analyze data obatined by HPLC; GraphPad Prism 9 (9.3.0) was used to analyze growth data; Excel 2016 (16.0.5305.1000) was used to analyze growth and metabolite concentration data.

For manuscripts utilizing custom algorithms or software that are central to the research but not yet described in published literature, software must be made available to editors and reviewers. We strongly encourage code deposition in a community repository (e.g. GitHub). See the Nature Portfolio guidelines for submitting code & software for further information.

Data

Policy information about availability of data

All manuscripts must include a data availability statement. This statement should provide the following information, where applicable:

- Accession codes, unique identifiers, or web links for publicly available datasets
- A description of any restrictions on data availability
- For clinical datasets or third party data, please ensure that the statement adheres to our policy

The atuhors declare that all data supporting the findings of this study are available wihtin the manuscript, its Supplementary Data and Source Data. Furthermore, the authors deposited the genome sequences in the NCDBI data base.

Field-specific reporting

Please select the one below that is the best fit for your research. If you are not sure, read the appropriate sections before making your selection.

☒ Life sciences ☐ Behavioural & social sciences ☐ Ecological, evolutionary & environmental sciences

For a reference copy of the document with all sections, see nature.com/documents/nr-reporting-summary-flat.pdf

Life sciences study design

All studies must disclose on these points even when the disclosure is negative.

Sample size	Sample-size was not a priori calculated. At least two biological duplicates and three technical triplicates were performed for each experiment and data had low variation between replicates compared to differences between sets of data from different experiments. For each type of experiment, sample size was chosen based on the common methods in the literature and based in our preliminary experiments.
Data exclusions	No data was excluded from the analysis.
Replication	All experiments were done in independent biological replicates performed on different days. The number of replicates are stated in each figure legend. All results were reproducible and no data set was excluded. All attempts of replication were successful.<br>For characterizing the editing window, biological duplicates, performed on different days, were used of each eight colonies (Figure 1E and F).<br>For determining position-dependent efficiency, biological duplicates, performed on different days, each containing 96 colonies were tested (Figure 2D). Determining multiplexing efficiency was performed in at least biological triplicates (Figure 2E). Fermentation experiments for production of protocatechuate was performed in biological triplicates (Figure 5B and C). Growth characterization of strain edited to prevent NADPH synthesis was performed in biological triplicates (Figure 6C, D, E and F).
Randomization	This study does not require the allocation of samples to different experimental groups. Thus, randomization was not necessary and was not performed.
Blinding	Blinding is not required for this study. Group allocation is not part of our experimental design. blinding during group allocation is irrelevant because the samples of bacterial cultures that were split into different conditions were random samplings and there is no control over which cells will be selected. Thus, there is no bias during group allocation.

Reporting for specific materials, systems and methods

We require information from authors about some types of materials, experimental systems and methods used in many studies. Here, indicate whether each material, system or method listed is relevant to your study. If you are not sure if a list item applies to your research, read the appropriate section before selecting a response.

Materials & experimental systems

n/a	Involved in the study	
☒	☐	Antibodies
☒	☐	Eukaryotic cell lines
☒	☐	Palaeontology and archaeology
☒	☐	Animals and other organisms
☒	☐	Human research participants
☒	☐	Clinical data
☒	☐	Dual use research of concern

Methods

n/a	Involved in the study	
☒	☐	ChIP-seq
☒	☐	Flow cytometry
☒	☐	MRI-based neuroimaging
