# Supplementary material for: Modular (de)construction of complex bacterial phenotypes by CRISPR/nCas9-assisted, multiplex cytidine base-editing
Source: Nat Commun. 2022 May 31;13:3026. doi: 10.1038/s41467-022-30780-z (PMC9156665; doi:10.1038/s41467-022-30780-z)
Supplement: Supplementary file 3 — Description of Additional Supplementary Files [file 41467_2022_30780_MOESM3_ESM.pdf]

Title: Supplementary Data 1.
Description: Python script for analyzing genomes for suitable protospacer towards base-editing to interrupt ORFs.

Title: Supplementary Data 2.
Description: Overview of SNPs found through WGS.

Title: Supplementary Data 3.
Description: Excel sheet with script for designing oligonucleotides for constructing base-editing plasmids.
